# Dietary Exposure Assessment of Veterinary Drug Residues in Animal-Derived Foods: Results from the Sixth Chinese Total Diet Study

**DOI:** 10.3390/foods15213905

**Published:** 2026-10-30

**Authors:** Yu Wei, Jun Ren, Chi Zhang, Jingguang Li, Dawei Chen

**Affiliations:** 1 NHC Key Laboratory of Food Safety Risk Assessment, Chinese Academy of Medical Science Research Unit (No. 2019RU014), China National Center for Food Safety Risk Assessment, Beijing 100021, China; 2 Inner Mongolia Institute for Drug Control and Research, Hohhot 010020, China; 3 School of Public Health, Jinzhou Medical University, Jinzhou 121001, China

**Keywords:** veterinary drug, total diet study, dietary exposure assessment, composite samples, animal-derived food

## Abstract

The global emergence of antibiotic residues in food-producing animals has raised substantial public health concerns due to their accumulation in edible tissues and organs. This investigation, conducted under the Sixth Chinese Total Diet Study (TDS), utilized ultra-high-performance liquid chromatography–high-resolution mass spectrometry to examine 96 composite dietary samples (derived from 1461 commonly consumed items) for the presence of 25 veterinary drugs (VDs) spanning seven therapeutic categories. The overall detection rate was 22.0%, with eggs (30.3%) and meats (30.0%) showing the highest rates. Four regulated VDs (trimethoprim, ofloxacin, enrofloxacin, and sarafloxacin), total sulfonamides, and three prohibited VDs (metronidazole, metronidazole, and chloramphenicol) in 22 samples (22.9%) exceeded the maximum residue limit (MRL) in China. These MRL comparisons are for reference only, as the MRL applicable to raw tissues does not match the cooked food composite samples analyzed in this study. Descriptive comparison across the Fourth to Sixth TDSs showed numerical differences in quinolone and sulfonamide detection rates and concentrations, though these findings require cautious interpretation due to methodological differences. For prohibited VDs lacking acceptable daily intakes (ADIs), the threshold of toxicological concern screening indicated that metronidazole and dimetridazole exceeded the genotoxic threshold, suggesting the need for further evaluation and attention. Notably, despite the high enrofloxacin occurrences (100% in aquatic products, 79.2% in meat samples), the estimated daily intakes for authorized VDs remained below 10% of their ADIs, indicating low health risks under the current exposure conditions.

## 1. Introduction

Veterinary drugs (VDs) are chemical compounds administered to animals for therapeutic purposes, including disease treatment and prevention. When used in compliance with good veterinary practices, they serve as essential tools in supporting the sustainable production of safe and sufficient animal-derived foods. This applies to key products, including meat, milk, eggs, and honey, and overall contributes to animal health and welfare [1]. The major public health concerns associated with VD residues encompass the emergence of antimicrobial resistance, hypersensitivity reactions, carcinogenicity, mutagenicity, teratogenicity, and disruption of the normal intestinal flora [2,3]. VD residues in animal-derived food primarily arise from three key sources: the improper and illegal application of pharmaceuticals; the inadequate observance of withdrawal periods; and environmental contamination [4,5]. An estimated 80% of food-producing animals receive antibiotic treatment at least once during their lifetime [6]. In response to the growing demand for animal and aquatic products, the majority of livestock and aquaculture species are reared under intensive farming systems [7,8,9]. Under such high-density conditions, animals experience significant physiological stress, which increases disease susceptibility. Consequently, authorized VD such as antibiotics and anthelmintics are routinely employed for disease prevention in these systems.

The Total Diet Study (TDS), implemented globally by national authorities, constitutes surveillance programs designed to assess food contamination levels and dietary exposure. Several nations have conducted TDSs investigating chemical compounds over recent decades, including the United Kingdom, Italy [10], United States [11], France [12], Canada [13], Australia [14], Cameroon [15], and Spain [16], with more recent initiatives undertaken in Germany [17], Japan [18], and China [19]. The TDS employs nationally representative dietary patterns from diverse geographical regions (commonly termed “market baskets”) to estimate the average intake of chemical compounds present in food across the general population [20]. This approach entails utilizing realistic contamination levels in foods as consumed at home (table-ready)to ensure accurate dietary intake risk assessments. Nevertheless, available data on VD residues remain limited. Comprehensive investigations are therefore needed to address this knowledge gap and alleviate potential public concerns regarding VD residues in animal-derived foods.

The Chinese TDS has been systematically conducted since 1990, with the seventh nationally representative survey underway as of 2023. Risk-based prioritization studies to support national residue monitoring programs remain limited in China, and the health implications of chronic low-level exposure to VDs are still inadequately characterized. To strengthen the evidence base, studies with longer durations and larger sample sizes are needed to assess VD residues and more accurately estimate associated consumer health risks. In response, the Sixth TDS implemented an integrated analytical strategy to enable simultaneous screening, quantification, and confirmation of multiple classes of VD residues. This approach leveraged complementary, high-throughput, and high-sensitivity detection techniques coupled with simple sample preparation to achieve efficient analysis. This study therefore aimed to fill a critical data gap in food safety risk assessment by investigating the occurrence of 25 VD residues from seven classes—namely, antibacterial synergists, quinolones, sulfonamides, imidazothiazoles, nitroimidazoles, benzimidazoles, and chloramphenicols—in four types of animal-derived foods using ultra-high-performance liquid chromatography–high-resolution mass spectrometry (UHPLC–HRMS). The specific objectives were three-fold: (i) to characterize the occurrence and concentration profiles of these VDs in the Sixth Chinese TDS; (ii) to assess chronic dietary exposure and health risk for the general Chinese adult population, using hazard quotients (HQs) for VDs with established or surrogate acceptable daily intakes (ADIs) and the threshold of toxicological concern (TTC) for genotoxic VDs without ADIs; and (iii) to descriptively compare the detection profiles with those from the Fourth and Fifth TDSs to explore potential temporal changes, but no statistical trend test was performed because of analytical methodological differences among the three TDS cycles.

## 2. Materials and Methods

### 2.1. Sample Preparation

Each province was designated as a distinct implementation unit. Depending on the population size, three to six survey points were established in each province. At each survey point, 30 households were randomly selected to participate in the food consumption survey. Data were collected through three consecutive days of household dietary records and 24-h recall interviews, which provided a representative overview of the average dietary patterns within each province. To formulate composite samples, a total of 5893 food raw materials were procured across 2016 to 2019 from 24 provincial-level administrative divisions (PLADs) in China [20]. These PLADs included Heilongjiang (HLJ), Liaoning (LN), Hebei (HeB), Beijing (BJ), Jilin (JL), Shanxi (ShX), Shaanxi (SX), Henan (HeN), Ningxia (NX), Inner Mongolia (NM), Qinghai (QH), Gansu (GS), Shanghai (SH), Fujian (FJ), Jiangxi (JX), Jiangsu (JS), Zhejiang (ZJ), Shandong (SD), Hubei (HuB), Sichuan (SC), Guangxi (GX), Hunan (HuN), Guangdong (GD), and Guizhou (GZ). This investigation, conducted for the first time at this scale, achieved unprecedented population coverage, representing 86% of China’s populace.

The collected food materials were classified into 13 distinct food categories. Based on locally prevalent cooking methods and utensils, the usage quantities were calculated during culinary preparation. This process ultimately yielded 288 composite samples for the Sixth TDS. Each composite sample represented one specific food category within one province. For each food category, representative food items were purchased from three or six sampling sites per province and mixed in equal proportions; the number of individual food items per composite varied by category (e.g., multiple vegetable types versus one or two egg types). The mixtures were cooked according to local dietary habits, with condiments added in calculated amounts, and then prepared using category-specific methods (e.g., homogenization for eggs; removal of inedible parts for meat and fish). Since VDs are primarily used during the breeding and transportation of food-producing animals, their residues are typically confined to animal-derived products. Consequently, a subset of 96 samples from the total 288 composite samples was selected for analysis. These samples represented four key animal-derived food categories: meats, eggs, aquatic foods, and dairy products derived from 1461 commonly consumed items (Table S1). All samples were preserved in a frozen state to establish a sample repository for evaluating exposure risks associated with emerging issues in subsequent TDS cycles. Detailed procedures for sample collection, cooking, and preparation are provided in the Supplementary Materials (Supplementary Text S1).

### 2.2. Detection Method

The selection of VDs for this study was guided by historical TDS monitoring data and preliminary screening results. The analytical scope included not only the parent compounds but also their relevant metabolites, as consumers may be exposed to both forms. A total of 34 analytes and their transformation products were targeted for analysis in the 96 composite samples. These analytes were categorized into 25 target VDs based on the residue definitions specified inGB 31650-2019 (National Food Safety Standard—Maximum Residue Limits for Veterinary Drugs in Foods) [21] and its supplementary standard GB 31650.1-2022 (National Food Safety Standard—Maximum Residue Limits for 41 Veterinary Drugs in Foods) [22]. For VDs whose residue definition is a sum of the parent compound and specified metabolites, the individual metabolite concentrations were first converted to parent-compound equivalents using molecular weight ratios (parent molecular weight/metabolite molecular weight), and the resulting equivalent concentrations were then summed. The resulting equivalent concentrations were then summed, and the result was expressed as the parent compound. This summed concentration was used for both reporting and dietary exposure estimation. The specific summation groups are detailed in the footnote of Table 1. The developed method enabled simultaneous identification and quantification of all 25 VD residues, achieving limits of detection (LODs) between 0.003 and 0.05 µg/kg while significantly reducing analytical time and operational costs (Table 1). It should be noted that cyfluthrin and fipronil, which also have veterinary applications, were excluded from this study as they were analyzed in an investigation focusing on pesticide residues. The analytical procedure used in the Sixth Chinese TDS (2016–2019) has been documented in detail in previously published studies [23,24,25]. In addition, we have provided a concise summary on the analytical method in the Supplementary Materials (Supplementary Text S2).

### 2.3. Dietary Exposure Assessment and Statistical Analysis

#### 2.3.1. Daily Food Consumption

A three-day survey design (two workdays plus one weekend day) was used to capture dietary differences between workdays and weekends. Each province served as an implementation unit, with three to six survey points per province and 30 randomly selected households per survey point. Household dietary data were collected using a weighing plus three-day accounting method. All raw and cooked foods, condiments, and drinking water were weighed or measured, and the quantities and varieties of all foods purchased each day were recorded. The consumption values from these four animal-derived categories, which were obtained from the sixth TDS [19,20,26], were shown in Table S2 in the Supplementary Information.

#### 2.3.2. Calculation of Dietary Exposure

Detected VDs were classified into three categories for risk assessment. For drugs with established ADIs, the estimated daily intake (EDI) was calculated and compared with the corresponding ADI to derive the HQ, defined as the percentage ratio of EDI to the corresponding ADI. For drugs without an established ADI but with suitable read-across data, a surrogate reference value was applied, and the HQ was derived. For genotoxic drugs without an established ADI (metronidazole, dimetridazole, and chloramphenicol), a TTC-based screening assessment was performed using the TTC value of 2.5 ng/kg bw/day for DNA-reactive genotoxic substances [27,28]. The EDI was compared with the TTC, and the EDI/TTC ratio was reported. An EDI exceeding the TTC indicates that the TTC screening is not passed, and the compound is identified as requiring further assessment or risk management.

Chronic dietary exposure to VD residues was assessed based on their actual concentrations in composite samples prepared in a table-ready form. The EDI for each VD was calculated using the following formula: EDI (μg/kg bw/day) = [c (μg/kg) × F (g/day)]/[1000 × BW (kg)], where c is the residue concentration (μg/kg), F is the daily food consumption (g/day), 1000 is the conversion factor from grams to kilograms, and BW is the adult mean body weight (63 kg). The body weight of 63 kg is the standardized “reference man” parameter used in the Chinese TDS, defined for atypical adult male aged 18–45 years who is engaged in light manual labor [20]. This parameter has been applied consistently since the first Chinese TDS in 1990 to ensure comparability of exposure estimates across different study cycles. Mean EDI was based on mean concentrations, whereas maximum EDI for worst-case screening was based on maximum concentrations; both used the same average daily consumption values. For chronic exposure assessment, mean concentrations were used as the primary input, while maximum concentrations were used only as a conservative reference for worst-case screening. For each VD, EDI was first calculated separately for meats, eggs, aquatic foods, and dairy products, and the four category-specific EDIs were then summed to obtain the total EDI. An HQ exceeding 100% was considered to indicate a potential health risk. All HQ values for this category were below 100%, indicating acceptable chronic dietary risk.

#### 2.3.3. Statistical Analysis

Statistical analyses were descriptive, except for one correlation analysis. Detection frequency (%) was defined as the percentage of samples with concentrations ≥ LOD. Mean and range values were calculated using the lower-bound approach: concentrations < LOD were assigned a value of zero; concentrations ≥ LOD but <LOQ were used as their estimated values provided by the analytical software; and concentrations ≥ LOQ were used as their actual measured values. Because of the composite sampling design, the limited number of detections for most compounds, and the single composite sample per food category per PLAD, no other statistical tests were performed. Comparisons across food categories, PLADs, and TDS cycles are therefore presented descriptively only. The only inferential analysis was Spearman’s rank correlation between trimethoprim and total sulfonamide residues, which was performed because the concentration data were non-normally distributed and contained a substantial proportion of zero values. Data processing was performed using SPSS 25.0 (IBM Corp., Armonk, NY, USA, 2017).

## 3. Results and Discussion

### 3.1. Occurrence Patterns and Concentration Levels of VDResidues

**Figure 1:**
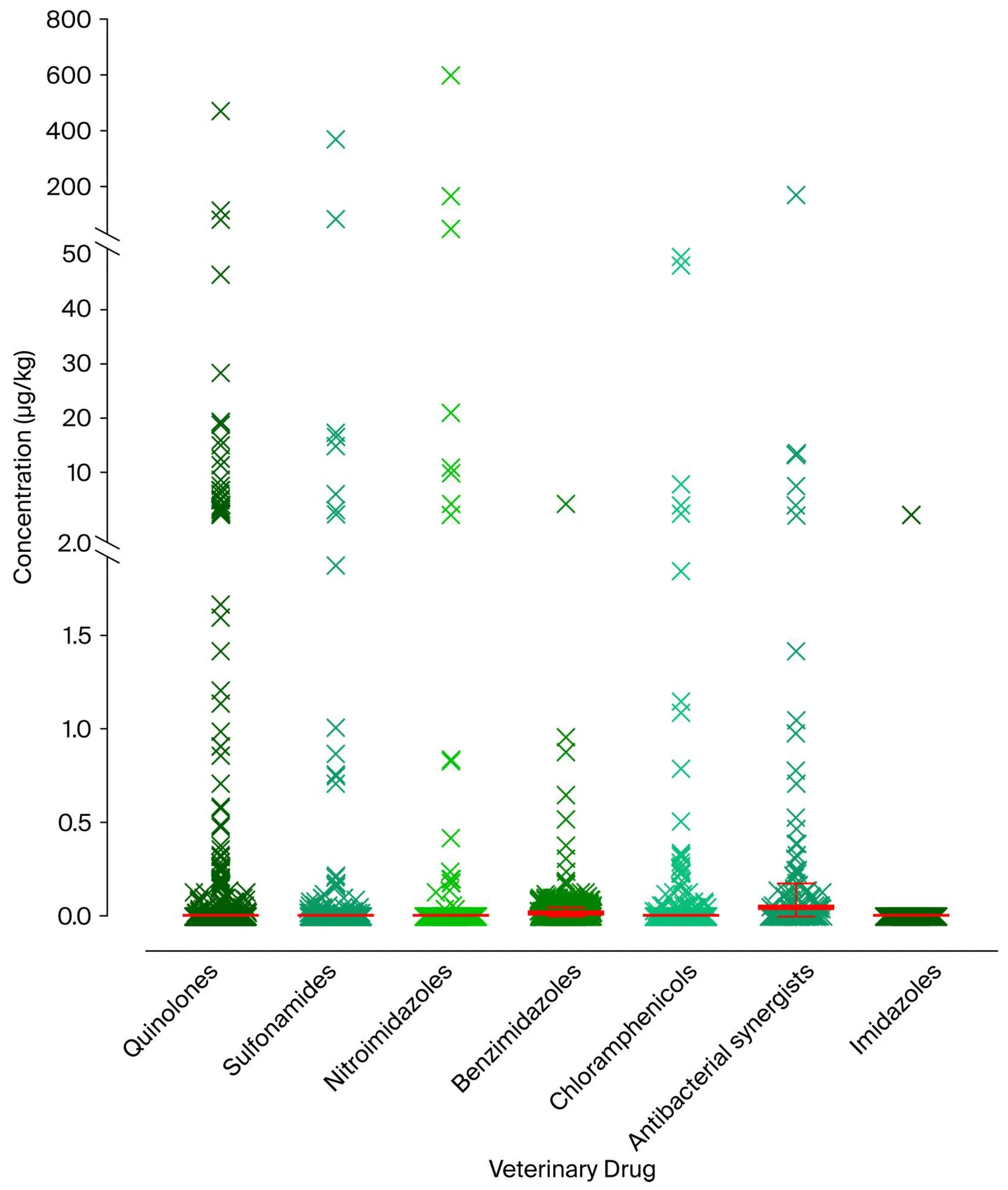
Scatter plot of the residue concentrations of VDs detected in the Sixth TDS. Data are presented as median with interquartile range.

The Sixth TDS represented the inaugural inclusion of several classes of VDs—namely, chloramphenicols (florfenicol), benzimidazoles (albendazole, fenbendazole, flubendazole, and mebendazole), antibacterial synergists, and imidazothiazoles (levamisole). Table 1 summarizes the detection frequencies and concentration ranges of these VDs in the composite samples. Twenty-five VDs were quantified, with concentrations ranging from 0.01 to 602.90 µg/kg, and the highest level was detected for metronidazole in eggs. Residue level analysis revealed that nitroimidazoles maintained the highest mean concentrations (4.56 ± 45.27 µg/kg), followed by antibacterial synergists (2.48 ± 17.78 µg/kg). Imidazothiazoles (0.047 ± 0.32 µg/kg) and benzimidazoles (0.050 ± 0.23 µg/kg) exhibited lower residue concentrations (Figure 1). Based on all VD-composite sample combinations (*n* = 2400), 1871 (78.0%) demonstrated residue concentrations below the LOD, while 55 (2.3%) contained detectable residues (concentrations ≥ LOD but <limit of quantification (LOQ)), and 474 (19.8%) contained quantifiable residues (concentrations ≥ LOQ). Among the 96 composite samples with detectable VD residues, 7 (7.3%) exhibited one residue, 83 (86.5%) contained two to nine residues, and 6 (6.3%) exceeded 10 residues. A maximum of 12 VDs were concurrently detected in two meat composite samples and one egg composite sample. Flubendazole (77.1%), fenbendazole (76.0%), mebendazole (68.8%), enrofloxacin (65.6%), and trimethoprim (54.2%) were the most frequently detected compounds in the 96 composite samples. These findings confirm that multiple VDs exist at low concentrations in the food chain. The increased frequency of VD detection may be attributed to improved method sensitivity rather than a temporary increase in drug use, although this possibility cannot be excluded.

### 3.2. Analysis of Samples with Residue Levels Above MRLs

The sampling was conducted between 2016 and 2019. GB 31650-2019 and GB 31650.1-2022 were published later and were not formally in force during most of the sampling period. However, for a more comprehensive and up-to-date assessment, these newer standards were adopted as the evaluation criteria. After excluding six compounds without established MRLs, 22 samples had 29 residue levels above current Chinese MRLs; these exceedances were indicative only (Table S3). The residues above MRLs comprised four regulated VDs—trimethoprim, ofloxacin, enrofloxacin, and sarafloxacin—along with three prohibited substances—metronidazole, dimetridazole, and chloramphenicol. Among the 22 samples with residues above MRLs, 16 were egg samples, 5 were meat samples, and 1 was a dairy product. A total of eight samples exceeded the MRLs for authorized VDs. Trimethoprim exceeded the limit in two egg samples; enrofloxacin exceeded the limit in three samples (two meat samples and one egg sample); ofloxacin exceeded the limit in two samples (one meat sample and one dairy product); and sarafloxacin exceeded the limit in one egg sample. In addition, when sulfonamides were calculated as total sulfonamide residues, three egg samples had residue levels above the MRL of 10 μg/kg. A total of 18 MRL-exceeding items involved prohibited substances: chloramphenicol in one meat sample; metronidazole in two meat and 13 egg samples; and dimetridazole in two egg samples. In total, 29 MRL-exceeding items were identified across 22 samples, with 7 samples containing 2 items each and 15 samples containing 1 item each.

Descriptive analysis showed VD residue levels in the samples exhibited a distinct non-normal distribution characterized by marked skewness and dispersion, along with several extreme values (Figure 1). The presence of these outliers, including samples with residue levels above MRLs, may reflect drug misuse or failure to observe mandatory withdrawal periods. The observed distribution aligns closely with typical VD use patterns in practical farming settings, such as intermittent administration, dosage inconsistencies, and individual animal variations.

### 3.3. Comparison of Concentrations of Residues by Food Category

**Figure 2:**
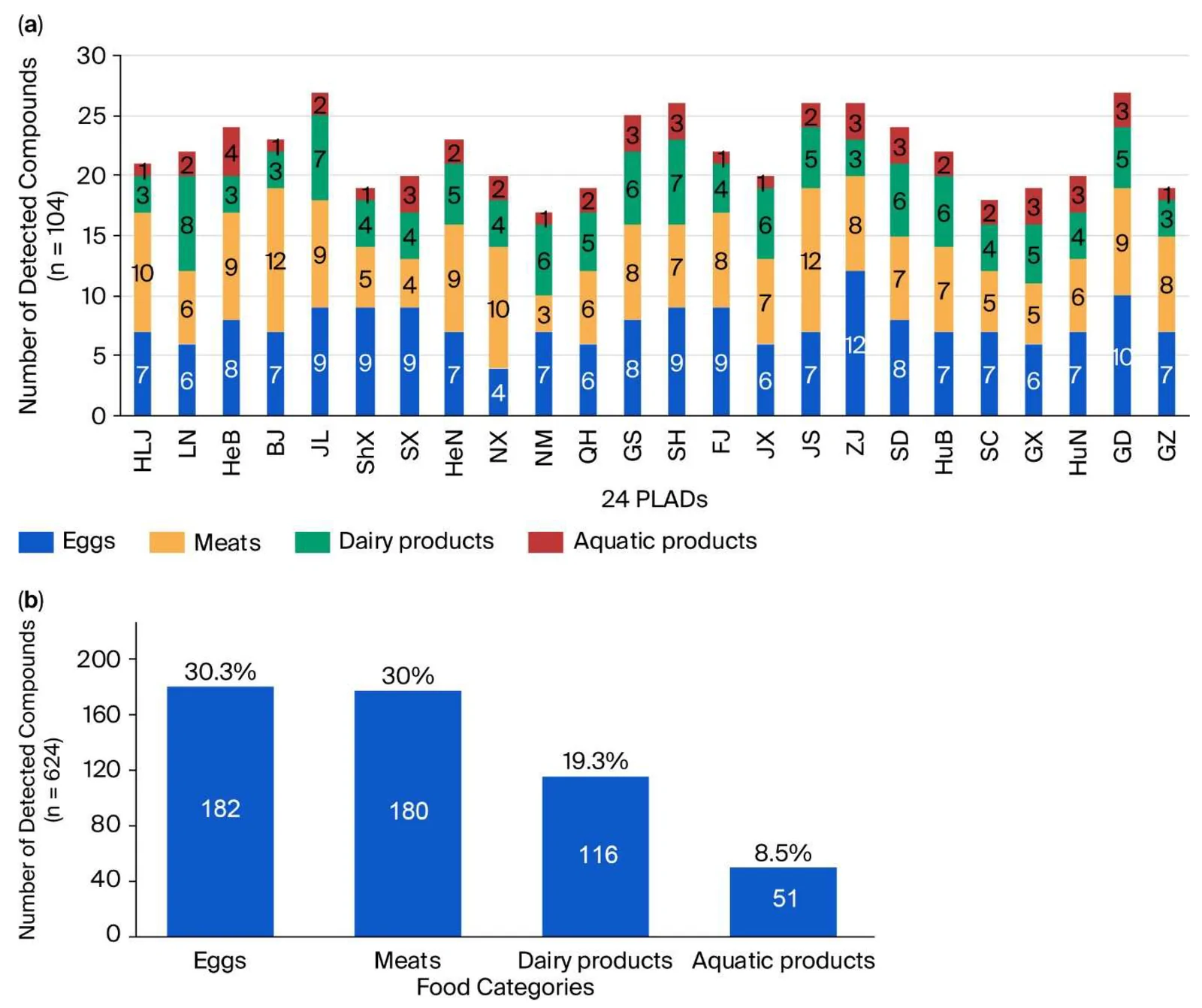
Regional comparison of VD detection frequenciesin four categories of animal-derived foods: (**a**) across all 24 PLADs; (**b**) within a single PLAD.

The overall detection rates of VD residues were highest in eggs (30.3%) and meats (30.0%), followed by dairy products (19.3%), with aquatic foods showing the lowest rate (8.5%) (Figure 2b). The concentration distributions of VD residues showed remarkable consistency between egg and meat samples: approximately 70% contained no detectable residues, 26% contained concentrations ≤1 µg/kg, 2% fell within the 1–10 µg/kg range, 1% contained 10–100 µg/kg, and fewer than 1% exceeded 100 µg/kg. In contrast, dairy and aquatic products exhibited lower residue levels. A total of 81% of dairy product samples contained no residues, 19% contained ≤1 µg/kg, and approximately 1% crossed the 1–10 µg/kg range. A total of 91% of aquatic food samples contained no residues, 5% contained ≤1 µg/kg, and 4% had higher ranges.

Eggs represent a fundamental component of the human diet and serve as an important protein source due to their high nutritional value, excellent bioavailability, and low cost. Under intensive farming conditions, high stocking densities increase poultry susceptibility to diseases, leading to the widespread use of VDs for growth promotion and infection prevention. These compounds are often not fully metabolized or excreted by hens and can accumulate in eggs, posing potential risks to consumer health. The elevated residue concentrations detected in eggs were primarily attributable to high levels of sulfamethoxazole, trimethoprim, metronidazole, and dimetridazole (>100 µg/kg) identified in samples originating from Shandong, Hunan, and Shanghai. Meanwhile, public perceptions regarding meat products demonstrate heightened sensitivity toward safety concerns, including regarding Bovine Spongiform Encephalopathy (BSE) [29], African Swine Fever (ASF) [30], contamination with dioxins and dioxin-like compounds [31], and hormone and VD residues [32]. Among all food commodities, consumer confidence decreases most markedly for meat products following safety incidents [33]. The significant role of animal-derived foods in the Chinese diet, attributed to their high nutritional value and palatability, underscores the critical importance of ensuring their safety and quality.

Traditional cooking methods, such as frying and baking, have been widely recognized in food safety research for their ability to reduce or degrade drug residues in foods due to the destabilizing effects of high-temperature treatment [34,35]. The effect of cooking on VD residues could not therefore be directly evaluated. The residue levels reported here reflect concentrations in foods as consumed, which may differ from those in raw commodities. This should be considered a limitation when comparing this study’s results with monitoring data based on raw agricultural products.

### 3.4. Statistical Analysis of Interrelationships Among VDs

Since the residue concentration data were non-normally distributed and contained a substantial proportion of zero values, Spearman’s rank correlation coefficient was used to examine the association between sulfonamides and antibacterial synergists. The statistically significant moderate positive correlation between trimethoprim and total sulfonamide residues (ρ = 0.690, *n* = 96, *p* < 0.001) was further supported by the synchronous detection of elevated concentrations of both compound classes in the same samples. For instance, in samples from Heilongjiang, trimethoprim (7.60 μg/kg) and sulfamonomethoxine (87.20 μg/kg) were co-detected; in samples from Jilin, trimethoprim (13.40 μg/kg) as detected with sulfamonomethoxine (6.09 μg/kg) and sulfamethoxazole (0.05 μg/kg); and in samples from Shandong, trimethoprim (173.15 μg/kg) was found alongside sulfamonomethoxine (1.01 μg/kg) and sulfamethoxazole (373.07 μg/kg). These findings are consistent with the common combined application of sulfonamides and trimethoprim in veterinary therapy, where their synergistic antibacterial effects are well recognized [36].

### 3.5. Comparison of Concentrations of Residues by Geographical Location

The analysis of VD residue concentrations across PLADs revealed distinct regional patterns. The highest detection rates were observed in Jilinand Guangdong, each registering 27.0% (27/100), while the lowest rate was documented in Inner Mongolia, at 17% (17/100) (Figure 2a). The mean residue concentrations varied across regions, with Hunan exhibiting the highest level (6.44 ± 60.28 μg/kg), followed by Shandong (5.49 ± 40.97 μg/kg), Beijing (5.01 ± 47.51 μg/kg), Shanghai (2.01 ± 17.01 μg/kg), Heilongjiang (1.69 ± 10.07 μg/kg), and Hebei (1.33 ± 11.79 μg/kg). Although descriptive statistics revealed certain regional variations in VD residue levels across the 24 PLADs—with some provinces showing higher mean concentrations or detection frequencies than others—these observed differences were descriptive only. There sidue levels of different compounds in samples from the same province or within the same composite sample may be correlated, and pooling would artificially inflate the sample size and produce unreliable statistical conclusions. Therefore, no inferential statistical test was performed. The observed variation may reflect random sampling variability rather than systematic geographical differences in VD use or farming practices. Furthermore, the observed residue patterns appeared to vary more markedly across food commodity types than across geographical regions. Eggs and meats generally showed higher detection rates and residue levels than aquatic foods and dairy products, whereas regional variations across provinces were less consistent. This observation had important implications for risk management: targeted monitoring efforts should continue to focus on high-risk food commodities, particularly eggs and meat, where residue levels were consistently higher than in aquatic foods and dairy products.

Among the 29 MRL-exceeding items identified, Jilin and Shanghai were the most affected regions, each with three such items. Hebei, Beijing, Inner Mongolia, Gansu, Fujian, Shandong, Hunan, and Guangdong each had two items with residue levels above MRLs, while Heilongjiang, Shanxi, Shaanxi, Henan, Zhejiang, Guangxi, and Guizhou each had one. Residue analysis revealed severe exceedances of regulatory limits, with metronidazole detected at 602.90 μg/kg in a Hunan sample, enrofloxacin measured at 475.10 μg/kg in a Beijing sample, and trimethoprim reaching 173.15 μg/kg in a Shandong sample. These MRL exceedances were indicative only and highlighted challenges in residue control; larger standard deviations were observed in the residue data from those regions (Figure 1). Therefore, it should be noted that, samples with residues above MRLs were detected in multiple provinces. This finding underscores the need for continued regulatory vigilance and enforcement, particularly in provinces where sporadic exceedances were identified. Collectively, these results suggest that while the national regulatory framework has been broadly effective in promoting the standardization of VD use across regions, commodity-specific risks and occasional residue levels above MRLs remain areas requiring sustained attention.

### 3.6. Assessment of Dietary Exposure to VDs

#### 3.6.1. HQ Assessment for VDs with Established or Surrogate ADIs

ADI values were obtained from GB 31650-2019 and GB 31650.1-2022, except for orbifloxacin, for which the ADI (0.012 mg/kg bw/day) was established by the Food Safety Commission of Japan based on the microbiological endpoint [37]. For pefloxacin, a read-across approach was applied using enrofloxacin as the structural analog because both share the same fluoroquinolone core structure and mechanism of action; the ADI of enrofloxacin was thus adopted as a surrogate reference value. For sulfonamides, because no individual ADIs are available, the ADI of sulfadimidine was used as a group reference, considering the shared sulfonamide core structure and common thyroid toxicity endpoint.

**Figure 3:**
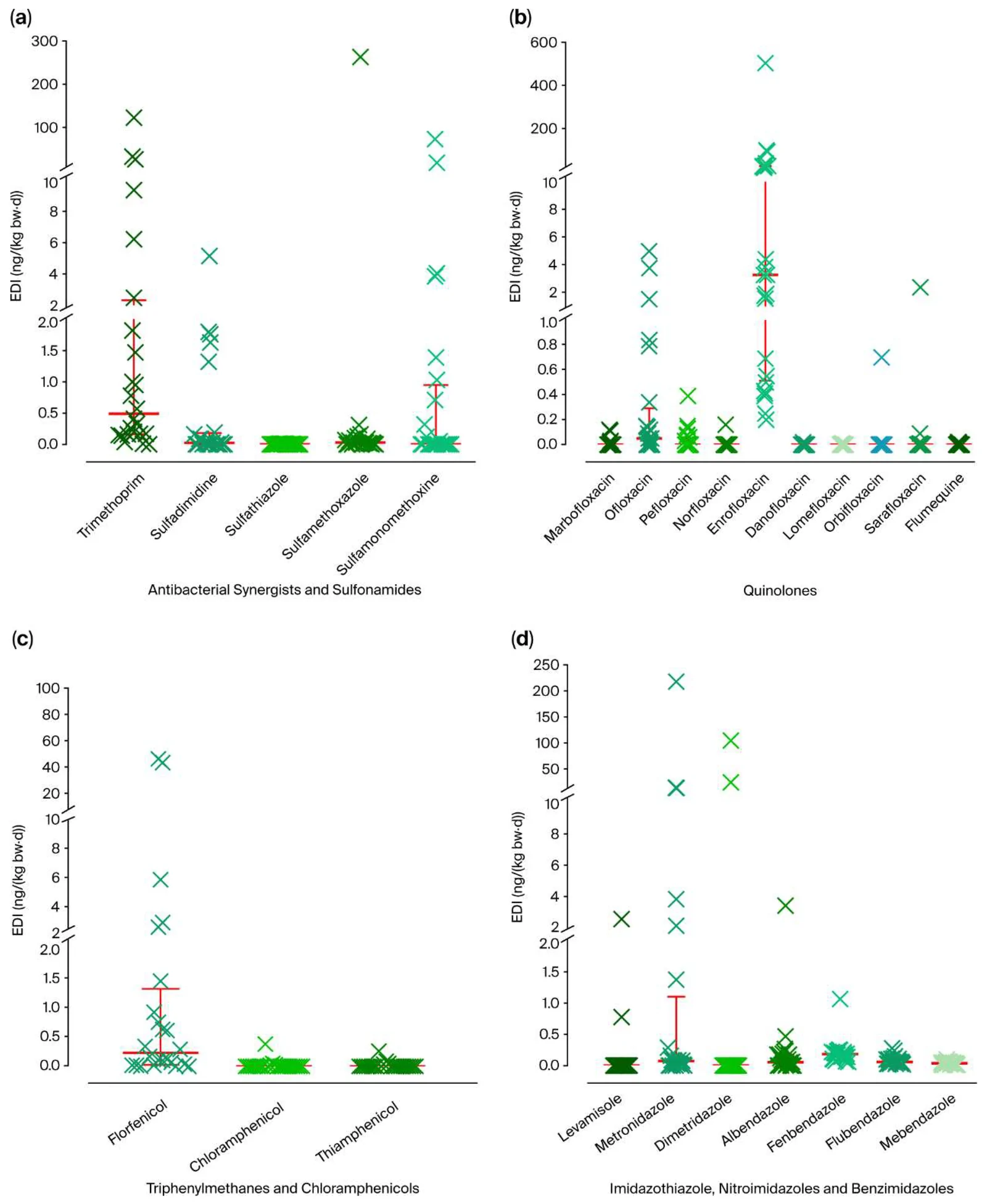
Scatter plot of the EDI ranges for VDs detected in the Sixth TDS: (**a**) antibacterial synergists and sulfonamides; (**b**) quinolones; (**c**) chloramphenicols; (**d**) imidazothiazoles, nitroimidazoles, and benzimidazoles. EDI, estimated daily intake. Data are presented as median with interquartile range.

Dietary exposures to VDs were evaluated by calculating the EDIs of detected residues and comparing these values to their corresponding ADIs. The average dietary intake of VDs ranged from 0.00063 to 35.35 ng/(kgbw·d) (Figure 3a–d), accounting for 0.0000025–0.57% of their ADIs (Table 2). Under the average adult consumption scenario, all HQs were below 10%, indicating a low health risk associated with dietary exposure to these residues in China. For VDs with established or surrogate ADIs, the calculated HQs for all detected VD residues remained below 100%, indicating that dietary exposure through animal-derived foods did not pose an unacceptable health risk to consumers under current exposure scenarios.

#### 3.6.2. TTC Assessment for Genotoxic VDs Without ADIs

For metronidazole and dimetridazole, the mean EDIs exceeded the genotoxic TTC by 4.24-fold and 2.15-fold, respectively. Based on the maximum EDIs, the exceedance was 87.28-fold and 41.91-fold (Table 3). Therefore, a potential health concern could not be excluded for these compounds, even under average dietary exposure. Chloramphenicol was detected at a maximum EDI of 0.38 ng/kg bw/day, which was below the TTC, but its presence in food was not permitted.

It should be noted that the TTC-based assessment can only indicate a potential concern; it cannot quantify the magnitude of risk. For metronidazole and dimetridazole, the TTC-based screening indicated that the mean EDIs exceeded the genotoxic TTC, meaning that a potential health concern could not be excluded. However, further quantitative risk characterization was not possible because these compounds lacked sufficient carcinogenicity data to derive a benchmark dose lower confidence limit for a 10% response (BMDL_10_). The analysis therefore yielded a screening alert, suggesting that these compounds required further regulatory attention.

### 3.7. Comparison of Three Consecutive TDS Datasets

Because the three TDS cycles differed in analytical methods, LODs, analyte lists, sample numbers, and provinces covered, the following comparison was descriptive and preliminary. A formal trend analysis using a common reporting limit and statistical tests requires further harmonization of the datasets and will be addressed in future work. The detection profiles were compared with those derived from the Fourth and Fifth TDSs to explore potential temporal differences. In the preceding surveys, liquid chromatography coupled with triple quadrupole mass spectrometry (LC-MS/MS) was employed for the identification and targeted quantification of VD residues. The LODs in the Sixth TDS were one to two orders of magnitude lower than those of the previous surveys. The higher reported detection rates of prohibited VDs in the Sixth TDS may be attributable, at least in part, to improved analytical sensitivity. The enhanced detection sensitivity and expanded monitoring capabilities helped identify residues that had previously been undetected or less frequently reported.

Although the overall dietary exposure to VDs in the Sixth TDS was numerically slightly higher than in the previous two surveys, several encouraging findings were observed. Most notably, neither β-receptor agonists nor β-receptor blockers, which are known to promote fat reduction and increase lean meat yield in animal production, were detected in any samples, suggesting that controls may have been effective over the misuse of these compounds. These positive developments may be attributable to the effectiveness of China’s enhanced food safety risk management measures. Several noteworthy residue concentration patterns were identified in the Sixth TDS. Trimethoprim, an antimicrobial synergist frequently administered with sulfonamides, was widely distributed across multiple food categories. Additionally, florfenicol showed relatively high detection rates specifically in meat and egg samples. Although VD residue levels showed certain numerical differences in the Sixth TDS compared with previous surveys, the overall pattern consistently indicated low exposure levels. A detailed summary of the EDI ranges of VDs across the Fourth, Fifth, and Sixth Chinese TDSs is presented in Table 4.

However, it is important to note that dietary exposure estimates from TDSs conducted indifferent countries are not directly comparable due to substantial differences in study design, analytical methodologies, and dietary habits. As methodological variations precluded meaningful direct comparisons, this analysis was therefore intentionally restricted to comparative assessments within the Chinese TDS framework, without attempting cross-national comparative evaluations. Indeed, the scarcity of TDS data on VD residues fundamentally limits the feasibility of conducting comparative studies.

### 3.8. Specific VD Residues in Composite Samples

#### 3.8.1. Quinolones

The analytical results demonstrate the presence of multiple quinolone residues, ranging from non-detectable to 475.10 μg/kg. The detection frequencies showed enrofloxacin as the most prevalent compound, at 65.6%, followed by ofloxacin (20.8%), pefloxacin (9.4%), flumequine (6.3%), sarafloxacin (5.2%), and marbofloxacin (4.2%), while danofloxacin and lomefloxacin were both detected at 2.1%, and norfloxacin and orbifloxacin at 1.0%. Enrofloxacin residues demonstrated high prevalence across the analyzed samples, with detection rates of 100% in aquatic products and 79.2% in meat samples.

**Figure 4:**
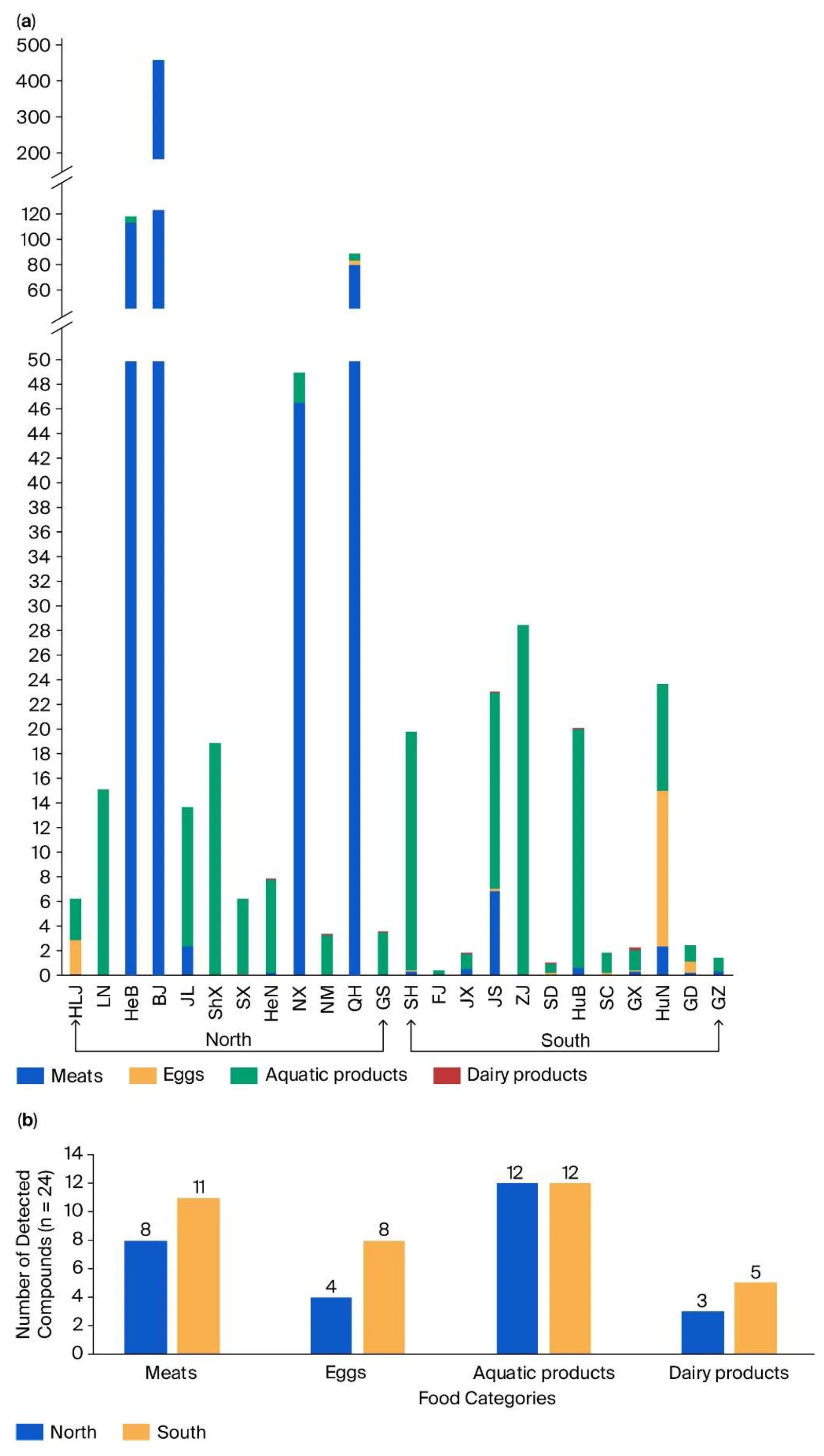
Detection profile of enrofloxacin in animal-derived foods from the Sixth TDS. (**a**) Four categories. (**b**) All 24 PLADs.

Enrofloxacin, a cost-effective synthetic broad-spectrum antimicrobial agent, is widely used in poultry farming for controlling fowl typhoid, mycoplasma gallisepticum infection, and pullorosis [40]. Chinese regulations explicitly prohibit its application in layer hens during the egg-laying period because of concerns that its residues could enter the human food chain, particularly through eggs. This study identified enrofloxacin residues exceeding the MRLs in two meat composite samples and one egg composite sample. The highest individual enrofloxacin concentrations in meat were observed in a few northern provinces, including Beijing (475.10 μg/kg), Hebei (117.81 μg/kg), Qinghai (84.41 μg/kg), and Ningxia (46.55 μg/kg) (Figure 4a). However, the overall distribution of enrofloxacin residues in meats were similar between northern and southern regions. In colder climates, poultry are more susceptible to respiratory and bacterial diseases, such as chronic respiratory disease and fowl typhoid, prompting farmers to apply antimicrobial agents more frequently as both treatment and prophylaxis. In contrast, enrofloxacin residues in most meat samples from southern provinces were below 10 μg/kg (Figure 4a). Nevertheless, this study’s data indicate that high enrofloxacin residue levels were sporadic rather than representative of a consistent regional difference. The detected residues in aquatic foods did not exceed 30 μg/kg, which suggests that current aquaculture management practices help mitigate residue risks. These findings highlight distinct regional and commodity-specific patterns in enrofloxacin residues and underscore the necessity for tighter controls on enrofloxacin use, particularly within northern China’s meat production systems, to mitigate potential health risks (Figure 4b).

Although ofloxacin and sarafloxacin were less frequently detected, residues above their MRLs were still observed. The levels of ofloxacin residues exceeded the limit in one meat sample from Beijing (4.10 μg/kg) and one dairy sample from Shanghai (3.12 μg/kg), indicating possible localized misuse or cross-contamination. Similarly, sarafloxacin residue levels above the MRL were detected in one egg sample from Gansu (6.78 μg/kg), suggesting inadequate monitoring of these drugs in certain production contexts. These results emphasize the need to strengthen monitoring and regulatory enforcement for quinolone residues, particularly in high-risk regions and commodity types.

Between the Fourth to Sixth TDSs, numerical differences were observed in quinolone residue levels in animal-derived foods. Residue concentrations were numerically higher, and the detection rate of quinolones was higher from 25.0% to 77.1%. Concurrently, the maximum recorded residue levels were also higher, from 106.32 μg/kg to 475.10 μg/kg, representing a greater than three-fold numerical difference [38,39]. The number of detected quinolone compounds was also larger from an initial 3 to 11. Notably, seven quinolones, including pefloxacin, were included and detected for the first time in this series in the Sixth TDS. Enrofloxacin, which showed the highest detection rate in the Sixth TDS, was first detected in the Fifth TDS. In contrast, ofloxacin and norfloxacin, both of which had been consistently detected since the Fourth TDS, showed lower reported residue levels over the same period. These descriptive patterns demonstrated that existing regulatory and intervention strategies had influenced VD usage practices, while simultaneously highlighting the pressing need to develop greener and more environmentally sustainable alternatives in VDs.

#### 3.8.2. Sulfonamides

Sulfonamides are broad-spectrum antibiotics known for their targeted efficacy and low cost [41]. As a result, they are widely used as feed or drinking water additives for disease prevention and treatment in livestock and poultry. In recent years, the successful development of novel, highly effective, long-acting sulfonamides and their synergistic antibacterial enhancers has further expanded their antibacterial spectrum, improved their persistence in the body, and reduced the risk of bacterial resistance [42]. These advances have significantly broadened the prospects for veterinary clinical applications. Sulfonamide detection rates were recorded as 31.3% for sulfamethoxazole, 14.6% for sulfadimidine, 12.5% for sulfamonomethoxine, and 7.3% for sulfathiazole. The residue concentrations ranged from 0.01 to 373.07 μg/kg for sulfamethoxazole, 0.02 to 3.11 μg/kg for sulfadimidine, 0.06 to 87.20 μg/kg for sulfamonomethoxine, and 0.02 to 0.04 μg/kg for sulfathiazole.

In China, MRLs have not been established for specific sulfonamides such as sulfadimidine, sulfamethoxazole, and sulfamonomethoxine, although a general MRL has been set for total sulfonamides residues. In this study, three egg samples were found to exceed the total sulfonamide MRL. These three samples were from Heilongjiang (87.20 μg/kg), Shandong (374.09 μg/kg), and Guizhou (15.15 μg/kg). The spatial variation in residue levels revealed distinct contamination profiles and highlighted region-specific pollution hotspots.

Between the Fourth and Sixth TDSs, residual levels of sulfonamides were numerically higher in the Sixth TDS. The detection rate was 4.2% in the Fourth TDS and 51.2% in the Sixth TDS. The maximum residue level was 124.57 μg/kg in the Fourth TDS and 374.09 μg/kg in the Sixth TDS. The number of detected sulfonamides was also larger. Sulfamethoxazole was consistently identified throughout all three TDS cycles, while sulfadimidine was first detected in the Fifth TDS and sulfamonomethoxine was newly detected in the Sixth TDS. Meat and egg products were identified as the primary sources of sulfonamide residues. In the Sixth TDS, the detection rates were 70.8% in meat samples and 83.3% in egg samples.

#### 3.8.3. Chloramphenicols

This study systematically analyzed the residue levels of chloramphenicols. The results showed that florfenicol residues ranged from 0.03 to 49.85 μg/kg, chloramphenicol from 0.03 to 0.23 μg/kg, and thiamphenicol from 0.06 to 0.27 μg/kg. The detection rates differed numerically (38.5%, 4.2%, and 4.2%), suggesting that florfenicol residues were more prevalent.

It should be noted that chloramphenicol is strictly regulated owing to its scientifically demonstrated serious health risks, including the potential to induce fatal bone marrow suppression [43]. Considering these risks, China had prohibited the use of chloramphenicol in food-producing animals to safeguard food safety and public health. Chloramphenicol was detected in some animal-origin food samples throughout three TDS cycles, with detection rates of 6.3% (3/48), 1.3% (1/80), and 4.2% (4/96), respectively. These findings indicate the persistence of the issue and reflect numerical differences in residue occurrence across different monitoring periods. Further analysis showed that chloramphenicol concentrations were detected in a relatively narrow range of 0.03–0.23 μg/kg, with most values near the limit of quantification, which suggested overall low residual levels.

Florfenicol, a widely used broad-spectrum antibiotic from the chloramphenicol class, has demonstrated efficacy against certain chloramphenicol-resistant bacterial strains, indicating its potential role in combating antimicrobial resistance. Studies showed that florfenicol exhibited stable chemical properties and a prolonged elimination half-life, allowing for extended retention in organisms [44]. In China, its use is strictly prohibited during the egg-laying period to ensure food safety but is permitted for disease prevention during the growth stage. In this study, florfenicol was detected in all four food categories, with an overall detection rate of 38.5%; the detection rates were 66.7% in eggs and 70.8% in meats. The residue concentrations ranged from 0.03 to 49.85 μg/kg in meats and 0.04 to 7.93 μg/kg in eggs, confirming the widespread presence of florfenicol residues. The residue levels in meat samples from Hubei (49.85 μg/kg) and Heilongjiang (48.28 μg/kg) were higher than those in other regions.

#### 3.8.4. Nitroimidazoles

Nitroimidazoles, including metronidazole and dimetridazole, are primarily used as antiprotozoal agents, with additional activity against anaerobic bacteria [45]. They are widely employed in veterinary medicine for the prevention and treatment of protozoal infections. Although their use in food-producing animals is prohibited in China, the residues of these drugs were still detected in this study, with detection rates of 18.8% and 2.1%, respectively. Eggs were identified as the primary source of nitroimidazole residues, with metronidazole showing a particularly high detection rate of 70.8% and a nationwide distribution. All dimetridazole-positive samples from the Sixth TDS were also derived from egg products, with no residues detected in other food categories. The highest residue levels were consistently found in egg samples, including metronidazole at 602.9 μg/kg in samples from Hunan, dimetridazole at 169.22 μg/kg in samples from Shanghai, and dimetridazole at 51.41 μg/kg in samples from Inner Mongolia. The higher detection frequency and concentration of nitroimidazole residues in eggs suggest potentially widespread unauthorized use within China’s poultry industry. From the Fourth to the Sixth TDSs, the detection rates for both compounds were numerically higher in the Sixth TDS. The detection rate of metronidazole was 2.1% in the Fourth TDS and 20.8% in the Sixth TDS, with positive samples primarily originating from eggs. Two dimetridazole-positive samples identified in the Sixth TDS were also traced to eggs from different regions. These differences could be attributed to either increased drug misuse in farming practices or enhanced monitoring capabilities, warranting sustained surveillance and regulatory attention.

#### 3.8.5. Imidazothiazoles and Benzimidazoles

Levamisole and benzimidazoles were included in the Sixth TDS for the first time in this series. In China, levamisole is prohibited for use during lactation and egg-laying periods, and is only permitted in limited quantities in meat products. However, two dairy product samples from Shanghai and Jiangxi were found to contain levamisole residues, both at a concentration of 2.25 μg/kg.

In the Sixth TDS, the targeted benzimidazoles included albendazole, fenbendazole, flubendazole, and mebendazole, which are commonly used anthelmintic agents in veterinary practice. Residue analysis showed that the residue levels of albendazole ranged from 0.02 to 4.29 μg/kg, with a detection rate of 21.9%. Fenbendazole showed residue levels between 0.02 and 0.52 μg/kg and were detected in 76.0% of samples. The residue levels of flubendazole ranged from 0.01 to 0.12 μg/kg, with a detection rate of 77.1%, while those of mebendazole ranged from 0.01 to 0.050 μg/kg, which was detected in 68.8% of the samples. The primary sources of these residues were identified as meat, eggs, and dairy products. Fenbendazole, flubendazole, and mebendazole were frequently detected across these three food categories, whereas their residues were rarely observed in aquatic products, likely due to limited use in aquaculture. Albendazole exhibited a lower detection frequency and was primarily found in meat products.

### 3.9. Limitations

This study had several limitations that warrant consideration.

#### 3.9.1. Tissue-Specific Distribution and Exposure Underestimation

VD residues, as significant environmental and food contaminants, demonstrate distinct tissue-specific distribution patterns. Residue concentrations typically accumulate preferentially in metabolically active organs such as the liver and kidneys, as well as in lipid-rich adipose tissues, primarily due to the lipophilic properties of many VDs and their reduced metabolic clearance rates in these tissues. In contrast, muscle tissues generally exhibit significantly lower residue levels. Since this investigation utilized composite meat samples consisting exclusively of muscle tissues while explicitly excluding organ meats, the obtained data likely underestimated the maximum exposure scenarios.

#### 3.9.2. Incomplete Coverage of VDs and Transformation Products

Despite substantial expansion in analytical coverage compared to previous TDSs, the methodological framework remained insufficient to monitor all potentially relevant VDs and their transformation products, particularly newly emerging compounds entering the market.

#### 3.9.3. Interpretation of MRL Exceedances in Composite Samples

The MRLs were established for specific raw tissues, whereas this study analyzed cooked, multi-species composite samples. The MRL exceedances reported here are therefore considered indicative only and should not be interpreted as direct regulatory violations. The composite sampling process could have diluted the high residue levels present in individual items, and cooking could have further altered the residue levels. These factors need to be considered when comparing this study’s results with regulatory limits.

#### 3.9.4. Temporal Variability in Sampling

The sampling for the Sixth TDS was conducted in batches between 2016 and 2019, which reflects the resource-intensive nature of the TDS and the logistical arrangements across provinces. Although the dietary survey followed a predefined seasonal design (spring for southern provinces and autumn for northern provinces), the collection of food samples was not restricted to a fixed season, and the specific sampling year and season varied across provinces. Consequently, temporal differences within each TDS cycle could not be completely ruled out. However, this study focused on comparing temporal differences across different TDS cycles rather than within a single cycle.

#### 3.9.5. Limited Availability of Toxicological Reference Values

The risk assessment relied heavily on established ADIs, which are unavailable for several frequently detected compounds, thereby constraining the comprehensive risk characterization of these compounds.

## 4. Conclusions

This study revealed that VD residues in Chinese animal-derived foods were characterized by widespread occurrence, diversity, and distinct regional patterns. Although dietary exposure risks for authorized VDs remained within acceptable limits, TTC screening for prohibited VDs without ADIs indicated that metronidazole and dimetridazole exceeded the genotoxic threshold (2.5 ng/kg bw/day), suggesting the need for further assessment and attention. The persistent detection of prohibited VDs, prevalent residues of unregulated drugs, and elevated concentrations in certain regions highlighted ongoing challenges. Descriptive comparison of the Fourth to Sixth TDSs showed that the reported detection frequencies and maximum concentrations of quinolones and sulfonamides were numerically higher in the Sixth TDS, and that the number of analytes included and detected was larger. While stringent national regulations appear to have controlled the use of high-risk drugs such as β-receptor agonists, substantial regulatory gaps persist for compounds that lack established maximum residue limits. The investigation identified particularly concerning residue patterns in northern China’s meat production systems and egg products, where elevated levels and frequent residue levels above MRLs were documented. Descriptive temporal comparison suggested possible changes in detection frequencies and residue levels for several major VD classes; however, these findings are preliminary and need to be interpreted with caution because the three TDS cycles differed in analytical methods, LODs, analyte lists, sample numbers, and provinces covered. These findings underscore the urgent need for enhanced regulatory frameworks, targeted monitoring programs, and the development of sustainable alternatives to conventional VDs in food animal production.

## Figures and Tables

**Table 1:** The detection frequencies and concentration ranges of VDs in 96 composite samples.

NO.	Veterinary Drugs	Limit of Detection (LOD) (µg/kg)	Limit of Quantification (LOQ) (µg/kg)	Number			Detection Rate	Concentration (µg/kg)	Max (µg/kg)
				X < LOD	LOQ > X ≥ LOD	X ≥ LOQ			
Antibacterial synergists							54.2%	2.48 ± 17.78	173.15
1	Trimethoprim	0.008	0.03	44	0	52	54.2%	2.48 ± 17.78	173.15
Quinolones							11.8%	1.01 ± 16.18	475.10
2	Marbofloxacin	0.02	0.06	92	1	3	4.2%	0.0085 ± 0.049	0.38
3	Ofloxacin	0.007	0.02	76	0	20	20.8%	0.13 ± 0.55	4.10
4	Pefloxacin	0.03	0.1	87	6	3	9.4%	0.0094 ± 0.036	0.27
5	Norfloxacin	0.01	0.03	95	0	1	1.0%	0.0033 ± 0.033	0.32
6	Enrofloxacin ^1^	0.01	0.03	33	6	57	65.6%	9.81 ± 50.54	475.10
7	Danofloxacin	0.01	0.03	94	0	2	2.1%	0.0018 ± 0.014	0.13
8	Lomefloxacin	0.02	0.06	94	2	0	2.1%	0.00073 ± 0.0055	0.050
9	Orbifloxacin	0.02	0.06	95	0	1	1.0%	0.025 ± 0.25	2.43
10	Sarafloxacin	0.01	0.03	91	1	4	5.2%	0.072 ± 0.69	6.78
11	Flumequine	0.01	0.03	90	2	4	6.3%	0.0020 ± 0.0084	0.060
Sulfonamides ^2^							16.4%	1.38 ± 19.59	373.07
12	Sulfadimidine	0.01	0.03	82	3	11	14.6%	0.087 ± 0.42	3.11
13	Sulfathiazole	0.01	0.03	89	6	1	7.3%	0.0017 ± 0.0063	0.040
14	Sulfamethoxazole	0.01	0.03	66	13	17	31.3%	3.90 ± 38.07	373.07
15	Sulfamonomethoxine	0.01	0.03	84	0	12	12.5%	1.53 ± 9.31	87.20
Imidazothiazoles							2.1%	0.047 ± 0.32	2.25
16	Levamisole	0.04	0.12	94	0	2	2.1%	0.047 ± 0.32	2.25
Nitroimidazoles							11.5%	4.56 ± 45.27	602.90
17	Metronidazole ^3^	0.05	0.15	78	3	15	18.8%	6.82 ± 61.53	602.90
18	Dimetridazole ^4^	0.05	0.15	94	0	2	2.1%	2.30 ± 18.00	169.22
Benzimidazoles							60.9%	0.050 ± 0.23	4.29
19	Albendazole ^5^	0.01	0.03	75	1	20	21.9%	0.093 ± 0.46	4.29
20	Fenbendazole ^6^	0.008	0.02	23	0	73	76.0%	0.066 ± 0.061	0.52
21	Flubendazole	0.003	0.01	22	0	74	77.1%	0.027 ± 0.024	0.12
22	Mebendazole	0.003	0.01	30	0	66	68.8%	0.015 ± 0.014	0.050
Chloramphenicols							15.6%	0.43 ± 4.11	49.85
23	Florfenicol ^7^	0.02	0.06	59	7	30	38.5%	1.27 ± 7.07	49.85
24	Chloramphenicol	0.02	0.06	92	2	2	4.2%	0.0039 ± 0.025	0.23
25	Thiamphenicol	0.03	0.1	92	2	2	4.2%	0.0053 ± 0.031	0.27

^1^ ∑Enrofloxacin = Enrofloxacin + Ciprofloxacin. ^2^ ∑Total sulfonamides = Sulfadimidine + Sulfathiazole + Sulfamethoxazole + Sulfamonomethoxine. ^3^ ∑Metronidazole = Metronidazole + Metronidazole-hydroxy. ^4^ ∑Dimetridazole = Dimetridazole + Dimetridazole-2-hydroxy. ^5^ ∑Albendazole = Albendazole sulphoxide +Albendazole sulphone + Albendazole 2-amino sulphone +Albendazole. ^6^ ∑Fenbendazole/Oxfendazole = Fenbendazole + Oxfendazole + Ofendazole sulfone. ^7^ ∑Florfenicol = Florfenicol + Florfenicol amine.

**Table 2:** Health risk indices of VD with established ADIs in composite samples from the Sixth TDS (*n* = 24).

Veterinary Drugs	EDIs (ng/(kgbw·d))		ADIs ^1^ μg/(kgbw·d)	HQs ^2^	
	Mean	Max		Mean	Max
Trimethoprim	8.61	122.32	4.2	0.21%	2.9%
Marbofloxacin	0.011	0.12	4	0.00028%	0.0030%
Ofloxacin	0.54	4.99	5	0.011%	0.10%
Norfloxacin	0.0068	0.16	14	0.000049%	0.0011%
Enrofloxacin	35.35	507.89	6.2	0.57%	8.2%
Danofloxacin	0.0010	0.023	20	0.0000050%	0.00012%
Lomefloxacin	0.00063	0.0076	25	0.0000025%	0.000030%
Sarafloxacin	0.10	2.39	0.3	0.033%	0.80%
Flumequine	0.0033	0.023	30	0.000011%	0.000077%
Sulfadimidine	0.51	5.15	50	0.0010%	0.010%
Levamisole	0.14	2.55	6	0.0023%	0.043%
Albendazole	0.24	3.42	50	0.00048%	0.0068%
Fenbendazole	0.21	1.07	7	0.0030%	0.015%
Flubendazole	0.082	0.28	12	0.00068%	0.0023%
Mebendazole	0.040	0.10	12.5	0.00032%	0.00080%
Florfenicol	4.41	45.93	3	0.15%	1.5%
Thiamphenicol	0.018	0.26	5	0.00036%	0.0052%
Orbifloxacin	0.029	0.70	12	0.00024%	0.0058%
Sulfamethoxazole	11.01	263.04	50	0.022%	0.53%
Sulfamonomethoxine	4.25	72.57	50	0.0085%	0.15%
Sulfathiazole	0.0029	0.015	50	0.0000058%	0.000030%
Pefloxacin	0.038	0.39	6.2	0.00061%	0.0063%

^1^ ADIs, the acceptable daily intakes. ^2^ HQs, Hazard quotients. HQ values are reported to two significant figures. EDI values ≥ 0.1 are reported to two decimal places, and those <0.1 to two significant figures.

**Table 3:** Health risk indices of VD without ADIs in composite samples from the Sixth TDS (*n* = 24).

VD	EDIs (ng/(kg bw·d))		TTC (ng/kg bw/d)	EDI ^1^/TTC ^2^		Interpretation
	Mean	Max		Mean	Max	
Metronidazole	10.59	218.19	2.5	4.24	87.28	Exceeds TTC; potential concern
Dimetridazole	5.38	104.78	2.5	2.15	41.91	Exceeds TTC; potential concern
Chloramphenicol	0.020	0.38	2.5	0.0080	0.15	Below TTC; detection is not permitted

^1^ EDI, estimated daily intake; ^2^ TTC, threshold of toxicological concern.

**Table 4:** Ranges of EDI for VDs across three consecutive Chinese TDSs.

Veterinary Drugs	EDI (ng/(kgbw·d))		
	Fourth TDS [38] (2007)	Fifth TDS [39] (2009~2013)	Sixth TDS (2016~2019)
β-receptor agonists and β-receptor blockers	0–7.46	0–1.24	* ^2^
Tetracyclines	0–2.86	0–0.59	*
Quinolones	0–72.2	2.22–119	0–507.89
Sulfonamides	0–84.6	0–3.01	0–263.04
Nitroimidazoles	0–20.8	0–120	0–218.19
Nitrofurans	0–2.06	0–2.06	/
Chloramphenicol	0–0.048	0–0.15	0–0.38
Florfenicol	/ ^1^	/	0–45.93
Levamisole	/	/	0–2.55
Benzimidazoles	/	/	0–3.42
Antibacterial synergist	/	/	0–122.32

^1^ “/”indicated that this work was not carried out. ^2^ “*”indicated that these drugs were not detected.

## Data Availability

The original contributions presented in the study are included in the article/Supplementary Materials, further inquiries can be directed to the corresponding authors.

## Supplementary Materials

The following supporting information can be downloaded at https://www.mdpi.com/article/10.3390/foods15213905/s1, Table S1: Number of survey sites, individual fooditems, and composite samples by PLAD. Table S2: Average daily consumption of 4 food categories for adults from 24 PLADs in the sixth TDS (g/day). Table S3: MRLs (µg/kg) for VDs in China and the number of samples with residue levels above MRLs identified in the 96 composite samples. Text S1. Total Diet Study in China. Text S2. Determination of Multi-Class Veterinary Drug Residues in Total Diet Study Samples.

## Abbreviations

The following abbreviations are used in this manuscript:

VD	Veterinary drug
TDS	Total Diet Study
UHPLC–HRMS	Ultra-high performance liquid chromatography–high resolution mass spectrometry
PLADs	Provincial-level administrative divisions
LOD	Limit of detection
EDI	Estimated Daily Intake
HQ	Hazard Quotient
ADI	Acceptable Daily Intake
LOQ	Limit of quantification
ASF	African Swine Fever
MRLs	Maximum Residue Limits
LC-MS/MS	Liquid chromatography coupled with triple quadrupole mass spectrometry
