# Supplementary material for: Dietary Exposure Assessment of Veterinary Drug Residues in Animal-Derived Foods: Results from the Sixth Chinese Total Diet Study
Source: Foods. 2026 Oct 30;15(21):3905. doi: 10.3390/foods15213905 (PMC13763346; doi:10.3390/foods15213905)
Supplement: Supplementary file 1 [file foods-15-03905-s001.zip › foods-4598166-supplementary.pdf]

Supplementary Material

Dietary Exposure Assessment of Veterinary Drug Residues in Animal-Derived Foods: Results from the Sixth Chinese Total Diet Study

Yu Wei 1,†, Jun Ren 1,2,†, Chi Zhang 1, Jingguang Li 1,*, and Dawei Chen 3,1,*

1 NHC Key Laboratory of Food Safety Risk Assessment, Chinese Academy of Medical Science Research Unit (No. 2019RU014), China National Center for Food Safety Risk Assessment, Beijing 100021, China; (Y. Wei) weiyu@cfsa.net.cn; (C. Zhang) zhangchi@cfsa.net.cn; (J. Li) lijg@cfsa.net.cn

2 Inner Mongolia Institute for Drug Control and Research, Hohhot 010020, China; (J. Ren) 58502015@qq.com

3 School of Public Health, Jinzhou Medical University, Jinzhou 121001, China; (D. Chen) dila2006@163.com

*Correspondence: (J. Li) lijg@cfsa.net.cn; (D. Chen) chendw@cfsa.net.cn

† These authors contributed equally to this work.

Text S1. Total Diet Study in China

S1. Dietary survey

S1.1 Respondents

The respondents of the dietary survey included households and individuals. Six survey sites for the provinces (municipality, autonomous region) with the population more than 50 million, and three survey sites for the provinceswith the population less than 50 million, were selected in TDS. Each survey site included 30 household.

S1.2 Selection of survey sites

A rural survey site is required to be rural areas with medium economic development which can represent the dietary habits of farmers in the province, while an urban survey site is required to be medium- to small-sized cities. The overall principle is that the survey sites shall be able to represent the general dietary habits, nutritional pattern and actual dietary structure of the residents of each province. The use of data about the economic status of local residents from statistical agencies can help make the selection more rational. The data collected from the survey sites in each province in the study were required to represent the average dietary structure of the province.

S1.3 Time of survey

In view of geographic and seasonal factors, as in previous studies, the survey was conducted in spring (from mid-April to late June) in the twelve southern provincesand fall (from September to late October) in the twelve northern provinces.

S1.4 Method of survey

There are often great dietary differences between workdays and the weekend, especially in cities. Therefore, in this dietary survey, the method of two workdays plus one weekend was adopted. For example: three days from the morning of Thursday (including breakfast) to the morning of Sunday (excluding breakfast), or from the morning of Sunday (including breakfast) to the morning of Tuesday (excluding breakfast).

S1.4.1 Household survey

The household-based dietary survey adopted a weighing plus three-day accounting method. The dietary survey of TDS attaches particular importance to the use of seasonings, and should therefore be based on on-site cooking of foods. From the first day of each survey session, all raw and cooked foods, condiments, beverages and drinking water were weighed or measured, where survey conductors would record the quantities and varieties of all foods purchased every day.

S1.4.2 Survey of individual respondents

The survey of individual respondents, which surveyed the dietary patterns of all family members, adopted 3-day 24-hour questioning plus registration. The survey of individual respondents was conducted simultaneously with the household survey. The survey method was the same as that used in the dietary survey of the national nutrition survey. On the second day of each survey session, survey conductors would ask each family member about the quantities of all foods consumed on the previous day and recorded them, including all raw and cooked foods, beverages and drinking water. Survey conductors would carefully check food items whose reported consumption by the whole family exceeded the amounts purchased to ensure the reliability of the survey results.

S1.5 Survey content

Survey content covered the varieties and quantities of all foods, drinking water and condiments consumed by the surveyed households or respondents and the ways of their use.

S1.6 Survey requirements

Survey conductors were required to help complete sheets and verify records, with particular attention given to the consumption of drinking water, beverages and condiments. In addition, survey conductors should cross-check the records to ensure data quality.

S1.7 Processing of dietary survey data

S1.7.1 Calculation of household dietary survey results

The results of the household dietary survey of each province were calculated to obtain the average consumption of each food category by each household and each adult male in the entire province (with no urban/rural differentiation) or the average consumption of each food category by each urban adult male and rural adult male in the province.

S1.7.2 Calculation of individual dietary survey results

The statistical calculation of individual dietary survey results was based on sex and age, respectively. The results were processed using the 3-day 24-hour recall method to obtain the per capital daily consumption of each food category by each age group.

S1.7.3 Determination of cooking methods of households and calculation of condiments used

The cooking methods of households followed the methods used by most subjects, which served as the basis for calculation of the actually used amounts of foods and condiments.

S2. Collection of food samples

S2.1 Classification of food samples

The per capital consumption of foods was classified into 13 categories according to the food composite approach adopted in this study, as shown in Table S1. Food items were cooked using quantities, methods, and kitchen utensils common to the locality, and condiments (thirteenth food category) were added to the other twelve sample categories at the calculated amount during the cooking procedure. This yielded the dietary structures of the regions or population groups in the study. If the foods consumed by a population group did not cover all thirteen categories, the missing categories were left blank.

S2.2 Food aggregation

The results of the household dietary survey were aggregated for calculation of average daily consumption of each food category by adult males in each province. The results of the individual dietary consumption were aggregated as per age groups for calculation of the daily consumption of each food category in each province, with the various food items being classified under their corresponding categories according to the principles and methods of aggregation. The purpose of aggregation is to effectively reduce the number of samples and the amount of work of sample collection, food cooking and processing and sample analysis without affecting the values of food consumption.

S2.3 Sampling

The sampling schemes were determined in the light of the amounts needed for sample analysis on

the basis of the varieties and quantities of food items in various food categories. Emphasis was placed on adequate preparation for the preparation and cooking of food samples. For example, vegetable washing ware, kitchen knives, chopping boards, weighing balances, and cooking sites and personnel were strictly managed to avoid sample decomposition and degradation caused by the delayed cooking of samples and its negative impact on the quality and progress of the study.

The samples of various food items were purchased at food purchase points such as vegetable markets, grocery stores, grain shops, farmer's markets and farmer homes in amounts slightly greater than the actually needed amounts according to the sampling scheme. Approximately one kilogram was purchased for each food item.

Food items purchased were required to be fresh, especially such food items as vegetables, fruits, meat, eggs, milk and fish, and grains should be free from impurities. Food samples were required to be taken to the cooking and processing sites as soon as possible and stored in the refrigerator at 4 °C if they could not be cooked immediately. Raw meat and fish products were stored in the freezing chamber for use.

S3. Cooking and sample preparation

S3.1 Food sample cooking

S3.1.1 Determination of food samples for cooking

The amounts of samples for cooking were calculated according to a standard man's consumption of various food categories and the amounts of samples needed for laboratory analysis. On this basis, a sampling sheet of food samples for cooking was prepared. Then, food items of each type collected from three sites were mixed equally to form the sample of the food type in the province, municipality or autonomous region in question, which would then be processed.

S3.1.2 Calculation of seasonings for cooking

According to the results of the dietary survey, various seasonings were put under various staple and non-staple food categories, and the amount of each seasoning to be used for the cooking of each food was calculated.

S3.1.3 Determination of cooking methods

The cooking methods of various foods were designed according to the recipes collected by the dietary survey and in the light of the local culinary habits and recipes.

S3.1.4 Cooking procedure

First, the various food samples and seasonings for cooking were processed according to applicable requirements and then cooked with cooking ware customarily used by local residents in the designated restaurant, kitchen or laboratory according to the designed cooking methods. Cooked foods or finished products that are directly edible did not need to be cooked. One difference in this cooking method from common cooking methods was that meat and vegetables were cooked separately, with post-cooking food samples and the amounts of water used for cooking weighed and recorded.

S3.2 Sample preparation

The various cooked food samples were prepared in their corresponding methods, as introduced briefly below.

1) Cooked food samples in the categories of cereals, soybeans, eggs, potatoes, and vegetables

were each evenly blended into homogenates.

2) Fruit samples collected from three points were weighed separately, mixed equally, and then had their peels, stones and inedible parts removed before the edible parts were weighed and blended into homogenates.

3) In the case of meat and fish products, inedible parts such as bones were removed before and after cooking, with other procedures the same as above.

4) For dairy products, fresh milk was cooked, and powdered milk was mixed with water (1 part powdered milk to 7 parts water, or as indicated by the product instruction) In brief, the powdered milk was mixed with cold boiled water to become diluted paste which was then further diluted by boiling water.

5) For sugars, brown sugar, white sugar and honey, if any, were not mixed but stored separately.

6) Beverages and water were prepared as follows: ① drinking water was boiled; ② tea water was prepared by mixing tea and boiling water of 70-80 °C (3 g tea to 250 mL water) for 5 min and then removing tea leaves. The drinking water ① in a room temperature environment was mixed with the tea water ② and/or other beverages in ratios according to their respective amounts of consumption.

7) For Alcohols, beer, fruit wine and white liquor were mixed in ratios based on their respective amounts of consumption.

8) For TDS in each province, various food samples were cooked and prepared according to the local dietary patterns and cooking methods, where 12 categories of food samples representing adult males and various age groups were composited, with the individual samples being stored.

S4. Transport and storage of samples

Samples were transported by air or train, preferably by air, ensuring that they would not unfreeze. The samples transported must be properly labeled, providing comprehensive information including source, bottling time, sample code, sample name, and quantity. Each sample bottle was required to bear two labels to avoid loss. All samples had to be stored at -20 °C for analysis.

Text S2. Determination of Multi-Class Veterinary Drug Residues in Total Diet Study Samples

Adapted from the China Total Diet Study SOP [23] *and the validated method of Fang et al.* [24].

S1. Purpose and Scope

This SOP describes the procedure for the determination of multi-class veterinary drug residues in composite diet samples from the China Total Diet Study. It is applicable to meat, eggs, aquatic products, milk.

S2. Principle

Samples are extracted with acetonitrile–water. After cold-induced acetonitrile–water phase separation, the extract is purified by dispersive solid-phase extraction (d-SPE) using anhydrous sodium sulfate, C18 and PSA. The purified extract is analyzed by ultra-high-performance liquid chromatography–high-resolution mass spectrometry (UHPLC–HRMS) on an Orbitrap Q Exactive instrument. Isotope-labelled internal standards are used for quantification. Full-scan/ddMS^2 and tSIM scan modes are used for screening and confirmation to ensure reliable and accurate results.

S3. Reagents and Materials

- Acetonitrile, methanol, formic acid: HPLC grade.
- Ultrapure water.
- Primary secondary amine (PSA) sorbent.
- C18 sorbent.
- Anhydrous sodium sulfate.
- Organic membrane filter: 0.22 µm.
- Amber glass containers, volumetric flasks, centrifuge tubes, autosampler vials, etc.

S4. Standards and Solutions

S4.1 Multi-veterinary drug stock solutions

Accurately weigh 10 mg (to 0.0001 g) of each veterinary drug standard. Dissolve in methanol and make up to 10 mL in a volumetric flask. Mix well and transfer to an airtight amber container. Store at −20 °C. Stable for 12 months.

S4.2 Mixed intermediate solution

Prepare a mixed intermediate solution at 100 mg/L in methanol. Store at −20 °C.

S4.3 Mixed working solution

Accurately transfer 0.1 mL of the 100 mg/L mixed intermediate solution into a 10-mL volumetric flask and make up to volume with methanol to obtain a 1 mg/L mixed working solution. Store at −20 °C. Stable for 3 months.

S4.4 Calibration/working standards

Prepare a series of mixed standard solutions in 75% acetonitrile/water. Transfer 3 mL of each solution into a 5-mL centrifuge tube, freeze at −20 °C for 1–2 h, and use the supernatant for injection.

S4.5 Internal standards

Add appropriate isotope-labelled internal standards before sample extraction. Use them for quantification and for monitoring method performance.

S5. Apparatus and Equipment

- UHPLC–HRMS system: Dionex U3000-Q Exactive HRMS (Thermo Fisher) or equivalent.
- Analytical balance: readability 0.1 mg.
- Ultrasonic generator.
- Vortex mixer.
- Centrifuge.
- Water bath.
- −20 °C and/or −80 °C freezer.
- 0.22 μm organic membrane filter.
- General laboratory glassware and plasticware.

S6. Sample Preparation

S6.1 Diet samples

a) Accurately weigh 5 g of the composite sample into a centrifuge tube.
b) Add an appropriate amount of isotope-labelled internal standard solution. Vortex and let stand for 30 min.
c) Add 0–4 mL water depending on the moisture content of the sample. Vortex to mix.
d) Add 15 mL acetonitrile and ultrasonicate for 30 min.
e) Centrifuge at 9500 rpm for 5 min at room temperature.
f) Transfer 3 mL of the supernatant into a 5-mL centrifuge tube and freeze at −20 °C for 1 h.
g) Transfer 1 mL of the supernatant into a 2-mL centrifuge tube containing 250 mg anhydrous sodium sulfate, 50 mg C18 and 50 mg PSA.
h) Vortex to mix and centrifuge at 15000 rpm for 5 min.
i) Filter the supernatant through a 0.22 μm organic membrane and transfer to an autosampler vial for UHPLC–HRMS analysis.

S6.2 Quality-control samples

For each batch, prepare at least one spiked recovery sample using a blank diet sample spiked at 10.0 μg/kg. A whole-procedure blank should also be processed in every batch: without weighing sample, carry out the complete sample preparation procedure, including internal standard addition, to verify that consumables and reagents do not introduce the target analytes.

S7. Instrumental Conditions

S7.1 Liquid chromatography

- Column: HSS T3, 1.8 μm, 2.1 mm × 100 mm.
- Column temperature: 40 °C.
- Injection volume: 5 μL.
- Flow rate: 300 μL/min.
- Mobile phase A: 0.1% formic acid in water.
- Mobile phase B: 0.1% formic acid in methanol.

Gradient program

Time (min)	Mobile phase A (%)	Mobile phase B (%)
0.0	98	2

Time (min)	Mobile phase A (%)	Mobile phase B (%)
4.0	80	20
5.5	60	40
10.5	0	100
12.9	0	100
18.0	98	2

S7.2 Mass spectrometry

Ion source: heated electrospray ionization (HESI), positive ion mode.

HESI temperature: 325 °C.

Capillary voltage: 3.8 kV.

Ion transfer tube temperature: 320 °C.

Sheath gas: 40 arb.

Auxiliary gas: 10 arb.

Acquisition mode: Full Scan/ddMS^2 (and tSIM when required).

Mass range: 100–1000 Da.

Automatic gain control (AGC): 3e6.

Maximum injection time: 100 ms.

Resolution: Full Scan 70,000 FWHM; ddMS^2 17,500 FWHM.

Normalized collision energy (NCE): 15, 35, 50%.

S8. Identification and Confirmation

Perform full-scan acquisition. Screen target compounds by comparing retention time and exact mass with the established standard database.

Screening criteria: retention time within ±0.2 min and mass error of the exact mass within 5 ppm → suspected positive; otherwise, negative.

According to EU Commission Decision 2002/657/EC [25], confirmation requires at least one precursor ion plus one product ion, or two product ions, matching the reference standard.

For suspected positive samples, further perform SIM-ddMS^2 or targeted MS/MS scanning for the suspected compound. Confirm the compound if at least two high-abundance fragment ions show a mass error <10 ppm compared with the reference spectrum and the relative ion abundance ratios are within the maximum permitted tolerances:

Relative ion abundance	Maximum permitted tolerance
>50%	±20%
>20–50%	±25%
>10–20%	±30%
≤10%	±50%

S9. Calculation

Calculate the veterinary drug content in the sample using the following equation:

$$X_i = \frac{c_i \times V \times f \times 1000}{m \times 1000}$$

Where

Xi = content of the veterinary drug in the sample, mg/kg.

ci = concentration of the veterinary drug in the final test solution, mg/L.

V = final dilution volume of the test solution, mL.

f = dilution factor.

m = sample mass, g.

Report the result as the arithmetic mean of two independent determinations obtained under repeatability conditions.

S10. Method Performance

Recovery: 60–120% for veterinary drugs in meat, eggs, aquatic products, and milk.

Precision: RSD ≤20%.

Repeatability: the absolute difference between two independent determinations under repeatability conditions shall not exceed 25% of their arithmetic mean.

LOD/LOQ: determined using matrix blank spiked solutions. LOD was defined as signal-to-noise ratio (S/N) = 3; LOQ was defined as S/N = 10.

Example recovery and precision data for eggs are given in *the validated data of Fang et al.* [24].

S11. Quality Control

A whole-procedure blank is processed in every batch, including internal standard addition, to check for contamination from reagents, consumables, and materials.

At least one spiked recovery sample is prepared per batch using blank diet sample spiked at 10.0 μg/kg.

Calibration checks, duplicate samples, and batch-to-batch QC are performed according to the China TDS quality-control protocol.

Internal standards are used for quantification and to monitor recovery and matrix effects.

Samples and extracts are stored at −20 °C or −80 °C before analysis. Storage stability is monitored as part of the TDS QC program.

12. References

[23] 23.Li, J.; Zhang, L.; Lv, B.; Chen, D.; Zhou, S. Standard Operation Procedure for Determination in China Total Diet Study; Science Press: Beijing, China, 2022. https://www.ecsponline.com/goods.php?id=222846

[24] Fang, C.; Gao, J.; Wang, Y.; Zhou, S.; Zhao, Y.; Chen, D.; Guo, Q. Determination of 125 veterinary drug residues in eggs by QuEChERS-ultra-high performance liquid chromatography-tandem mass spectrometry. Chin. J. Chromatogr. 2018, 36, 1119–1131.

[25] 2002/657/EC: Commission Decision of 12 August 2002 implementing Council Directive 96/23/EC concerning the performance of analytical methods and the interpretation of results (Text with EEA relevance) (notified under document number C(2002) 3044). Official Journal L 221 , 17/08/2002 P. 0008 – 0036. http://data.europa.eu/eli/dec/2002/657/oj

Table S1. Number of survey sites, individual food items, and composite samples by PLAD.

PLADs	Number of survey sites	Number of individual samples				Number of composite samples
		Meats	Eggs	Dairy products	Aquatic foods	
Sixth TDS	103	621	222	218	400	96
Heilongjiang	3	24	9	9	6	4
Jilin	3	15	6	3	6	4
Liaoning	3	18	6	6	18	4
Beijing	3	28	6	6	12	4
Shanxi	3	18	3	9	6	4
Hebei	6	36	12	18	18	4
Henan	6	30	6	6	30	4
Ningxia	3	18	3	6	9	4
Shaanxi	3	18	6	3	6	4
Inner Mongolia	3	9	3	6	6	4
Qinghai	3	15	3	6	6	4
Gansu	3	15	6	9	6	4
Fujian	3	18	9	6	18	4
Zhejiang	6	36	18	12	30	4
Jiangsu	6	42	12	12	30	4
Shanghai	6	48	12	18	42	4
Jiangxi	3	15	6	9	6	4
Shandong	6	36	12	12	30	4
Hubei	6	36	18	12	30	4
Sichuan	6	36	12	12	6	4
Hunan	6	42	18	12	30	4
Guangxi	3	15	9	6	15	4
Guangdong	7	35	21	14	28	4
Guizhou	3	18	6	6	6	4

Abbreviations: PLADs=provincial-level administrative divisions; TDS=Total Diet Study.

Table S2. Daily food consumption of 4 food categories for adults from 24 PLADs in the sixth TDS (g/day).

Food categories	North 1						North 2					
	**HLJ**	**LN**	**HeB**	**BJ**	**JL**	**ShX**	**SX**	**HeN**	**NX**	**NM**	**QH**	**GS**
Meats	548.0	615.5	755.3	795.1	585.5	1037.6	762.6	924.2	661.5	723.0	766.2	686.2
Eggs	40.5	40.9	31.5	38.8	40.2	21.0	22.0	29.7	13.0	29.7	13.1	22.2
Dairy products	27.2	16.1	9.5	16.4	11.1	6.4	4.1	4.6	2.4	10.3	3.6	2.8
Aquatic products	62.8	76.9	37.7	113.6	87.8	34.9	37.9	24.2	84.6	113.0	15.6	33.2
**Food categories**	**South 1**						**South 2**					
	**SH**	**FJ**	**JX**	**JS**	**ZJ**	**SD**	**HuB**	**SC**	**GX**	**HuN**	**GD**	**GZ**
Meats	449.1	632.3	629.9	778.0	632.6	995.7	528.3	969.8	940.6	577.4	456.6	674.9
Eggs	39.0	19.0	20.5	28.1	23.9	44.4	25.0	14.6	14.0	22.8	23.9	16.3
Dairy products	68.6	64.2	43.7	39.0	50.3	22.8	40.7	8.4	96.7	66.4	51.2	3.5
Aquatic products	73.4	59.8	56.7	27.2	67.3	54.5	19.2	14.9	64.4	63.4	29.3	20.9

Abbreviations: Heilongjiang (HLJ), Liaoning (LN), Hebei (HeB), Beijing (BJ), Jilin (JL), Shanxi (ShX), Shannxi (SX), Henan (HeN), Ningxia (NX), Inner Mongolia (NM), Qinghai (QH), Gansu (GS), Shanghai (SH), Fujian (FJ), Jiangxi (JX), Jiangsu (JS), Zhejiang (ZJ), Shandong (SD), Hubei (HuB), Sichuan (SC), Guangxi (GX), Hunan (HuN), Guangdong (GD), Guizhou (GZ).

Table S3. MRLs (μg/kg) for VDs in China and the number of samples with residue levels above MRLs identified in the 96 composite samples.

Food categories		Trimethoprim	Ofloxacin	Pefloxacin	Norfloxacin	Enrofloxacin	Danofloxacin	Lomefloxacin
Muscle	MRLs[1]	100/50	2	2	2	100	200/100	2
	Number[2]	0	1	0	0	2	0	0
Milk	MRLs	50	2	2	2	100	30	2
	Number	0	1	0	0	0	0	0
Fish (skin and flesh)	MRLs	50	2	2	2	100	100	2
	Number	0	0	0	0	0	0	0
Egg	MRLs	10	2	2	2	10	10	2
	Number	2	0	0	0	1	0	0

Food categories		Sarafloxacin	Flumequine	Sulfadimidine	Levamisole	Metronidazole	Dimetridazole	Albendazole
Muscle	MRLs	10	500	100	10	Banned	Banned	100
	Number	0	0	0	0	2	0	0
Milk	MRLs	/[3]	50	25	/	Banned	Banned	100
	Number	/	0	0	/	0	0	0
Fish (skin and flesh)	MRLs	30	500	100	/	Banned	Banned	/
	Number	0	0	/	/	0	0	/
Egg	MRLs	5	10	10	5	Banned	Banned	/
	Number	1	0	0	0	13	2	/

Food categories		Fenbendazole	Flubendazole	Florfenicol	Chloramphenicol	Total sulfonamides	
Muscle	MRLs	100/50	200/10	300/200/100	Banned	100	
	Number	0	0	0	1	0	
Milk	MRLs	100	/	/	Banned	100	
	Number	0	/	/	0	0	/
Fish(skin and flesh)	MRLs	/	/	1000	Banned	100	
	Number	/	/	0	0	0	
Egg	MRLs	1300	400	10	Banned	10	
	Number	0	0	0	0	3	

[1]MRLs, Maximum Residue Limits. [2]Number, Number of non-conforming samples. [3]"/", absence of MRLs
